# Comparison of the efficacies of first-generation epidermal growth factor receptor tyrosine kinase inhibitors for brain metastasis in patients with advanced non-small-cell lung cancer harboring EGFR mutations

**DOI:** 10.1186/s12885-018-4911-7

**Published:** 2018-10-22

**Authors:** Naoto Aiko, Tsuneo Shimokawa, Kazuhito Miyazaki, Yuki Misumi, Yoko Agemi, Mari Ishii, Yukiko Nakamura, Takeharu Yamanaka, Hiroaki Okamoto

**Affiliations:** 1 Department of Respiratory Medicine, Yokohama Municipal Citizen’s Hospital, 56 Okazawa-cho, Hodogaya-ku, Yokohama-city, Kanagawa 240-8555 Japan; 2 Department of Medical Oncology, Yokohama Municipal Citizen’s Hospital, 56 Okazawa-cho, Hodogaya-ku, Yokohama-city, Kanagawa 240-8555 Japan; 3 Department of Biostatistics, Yokohama City University School of Medicine, 3-9 Fukuura, Kanazawa-ku, Yokohama-city, Kanagawa 236-0004 Japan

**Keywords:** Brain metastasis, EGFR TKI, Erlotinib, Gefitinib, Lung cancer, NSCLC

## Abstract

**Background:**

Compared with standard chemotherapy, epidermal growth factor receptor tyrosine kinase inhibitors (EGFR-TKIs) are more effective in patients with advanced non-small-cell lung cancer (NSCLC) harboring EGFR mutations. However, data comparing the efficacies of different EGFR−TKIs, especially regarding the presence of brain metastasis, are lacking.

**Methods:**

EGFR-TKI naive patients with recurrent or stage IIIB/IV NSCLC harboring EGFR mutations, excluding resistance mutations, were enrolled in this study. We retrospectively determined progression-free survival (PFS) using the Kaplan−Meier method with log-rank test in patients treated with either gefitinib or erlotinib, cumulative incidence of central nervous system (CNS) progression using the Fine and Gray competing risk regression model, and favorable prognostic factors for CNS progression by multivariate analysis.

**Results:**

Seventy-seven EGFR-TKI-naive patients were started on either gefitinib (*n* = 55) or erlotinib (*n* = 22) in our hospital from April 2010 to April 2016. Among the patients with brain metastasis, PFS tended to be longer in the erlotinib than in the gefitinib group. In the analysis of cumulative incidence, the probability of CNS progression was lower in the erlotinib group than in the gefitinib group. Particularly, in a subgroup analysis of the patients with brain metastasis, there was a significant difference between the erlotinib and gefitinib groups (hazard ratio 0.25; 95% confidence interval, 0.08–0.81; *p* = 0.021). Of the prognostic factors for CNS progression evaluated, the absence of brain metastasis before EGFR-TKI therapy and receiving erlotinib (vs gefitinib) had a significantly favorable effect on patient prognosis.

**Conclusion:**

Although this was a retrospective analysis involving a small sample size, erlotinib is potentially more promising than gefitinib for treatment of brain metastasis in patients with EGFR-mutant NSCLC.

## Background

Approximately 40% of patients with non-small-cell lung cancer (NSCLC) develop brain metastasis during the course of their disease [1]. And the risk of brain metastasis is greater in patients harboring epidermal growth factor receptor (EGFR) mutations [2].

Compared with standard chemotherapy, EGFR tyrosine kinase inhibitors (EGFR-TKIs) are more effective in patients with advanced NSCLC harboring EGFR mutations [3–6]. Several case reports and studies involving small patient series indicated successful treatment of brain metastasis using EGFR-TKIs [7–10]. However, few studies have compared individual EGFE-TKIs in terms of their efficacy against brain metastasis.

The aim of our study was to evaluate retrospectively the effect of two first-generation EGFR-TKIs (gefitinib and erlotinib) on brain metastasis in patients with NSCLC harboring EGFR mutations.

## Methods

### Patient selection

Patients were chosen from the medical records of Yokohama Municipal Citizen’s Hospital if they were recurrent or stage IIIB/IV NSCLC harboring EGFR mutations excluding resistance mutations and received either gefitinib or erlotinib based on physicians’ choice for the first EGFR-TKI treatment. The other criteria included Eastern Cooperative Oncology Group performance status (ECOG-PS) ranging from 0 to 3, presence of measurable disease, and adequate organ functions. The exclusion criteria were active infection, uncontrolled angina, myocardial infarction in the previous 6 months, uncontrolled hypertension and diabetes mellitus, interstitial pneumonitis and lung fibrosis as identified on a chest x − ray, severe mental disorders, and pregnant or lactating women. For assessment disease stage, all patients underwent computed tomography (CT) of the thorax and upper abdomen, either CT or magnetic resonance imaging (MRI) of the brain, and either radioisotopic bone scan or positron emission tomography (PET). CT was basically repeated every 6–8 week to evaluate the target lesions. Tumor response was assessed using the Response Evaluation Criteria in Solid Tumors version 1.1. The study population was assessed using the tumor, node, metastasis staging system (seventh edition of the American Joint Committee on Cancer staging manual).

### Statistical analyses

Progression free survival (PFS) was defined as the interval from the start of EGFR-TKI treatment to disease progression or death from any cause. Alive without progression (data cutoff date, October 31, 2016) and loss to follow-up were censored. PFS was analyzed using the Kaplan-Meier method and compared using the log-rank test. The Fine and Gray competing risk regression model was used to compare cumulative incidence of central nervous system (CNS) progression between gefitinib and erlotinib. Death without CNS progression was considered a competing risk in the analysis, and alive without CNS progression (data cutoff date, October 31, 2016) and loss to follow-up were censored. CNS progression was confirmed by brain MRI or contrast-enhanced CT. In the subgroup-analysis, we analyzed the PFS and cumulative incidence of CNS progression in patients who had brain metastasis before EGFR-TKI administration and those who did not.

The prognostic factors for CNS progression evaluated were age at initiation of EGFR-TKI administration, sex, ECOG PS, presence or absence of brain metastasis before starting EGFR−TKI treatment, and type of EGFR-TKI (gefitinib or erlotinib). Multivariate analysis of the favorable prognostic factors of CNS progression was conducted using the Cox proportional hazards model.

A *P*-value < 0.05 was considered to indicate a statistically significant difference. All analyses were performed using STATA 14.

## Results

### Patient characteristics

The patient characteristics are shown in Table 1. In total, 77 patients with NSCLC harboring EGFR mutations were enrolled in this study. Of these, 55 and 22 patients received gefitinib and erlotinib, respectively, as first EGFR-TKI treatment. More patients had poor ECOG PS (≧2) in the gefitinib group (16 [29%]) compared with erlotinib group (4 [18%]). Gefitinib (44 [80%]) was administered as first-line therapy more frequently than erlotinib (9 [41%]). As for brain metastasis, more of patients who treated with erlotinib have had brain metastasis (12 [55%]) and received radiation therapy (6 [27%]) prior to EGFR-TKI treatment compared with those treated with gefitinib. No patient who received surgery for brain metastasis and immune check point inhibitor therapy prior to EGFR-TKI was included in both group.

**Table 1:** Patient characteristics

	Gefitinib (*n* = 55)	Erlotinib (*n* = 22)
Sex, n (%)		
Male	19 (35)	11 (50)
Female	36 (65)	11 (50)
Median age, years (range)	71 (46–91)	71 (47–83)
ECOG PS, n (%)		
0–1	39 (71)	18 (82)
≧2	16 (29)	4 (18)
TNM stage, n (%)		
3B	3 (5)	1 (5)
4	43 (78)	16 (73)
Recurrence	9 (16)	5 (23)
Previous chemotherapy regimen, n (%)		
0	44 (80)	9 (41)
1	9 (16)	7 (32)
≧2	2 (4)	6 (27)
Brain metastasis, n (%)		
0	40 (73)	10 (45)
≧1	15 (27)	12 (55)
Radiotherapy for brain metastasis before EGFR-TKI treatment, n (%)	4 (7)	6 (27)
WBRT	1 (2)	1 (4)
SRT	3 (5)	5 (23)
EGFR mutation, n (%)		
Exon19 del	24 (44)	6 (27)
Exon21 L858R	29 (53)	15 (68)
Minor	2 (4)	1 (5)
Dose reduction or intermittent administration, n (%)	13 (24)	10 (45)
Best response, n (%)		
CR	0 (0)	0 (0)
PR	24 (44)	11 (50)
SD	20 (36)	9 (41)
PD	4 (7)	1 (5)
Unknown	7 (13)	1 (5)
The reason of EGFR-TKI discontinuation, n (%)		
Disease progression	36 (65)	17 (77)
CNS progression	10 (18)	2 (9)
Adverse event	5 (9)	2 (9)
Other	7 (13)	1 (5)
Ongoing	7 (13)	2 (9)

*ECOG PS* Eastern Cooperative Oncology Group performance status, *WBRT* whole brain radiotherapy, *SRT* stereotactic radiotherapy, *CR* complete response, *PR* partial response, *SD* stable disease, *PD* progressive disease, *CNS* central nervous system, *EGFR-TKI* epidermal growth factor receptor tyrosine kinase inhibitor

### Progression free survival

**Fig. 1:**
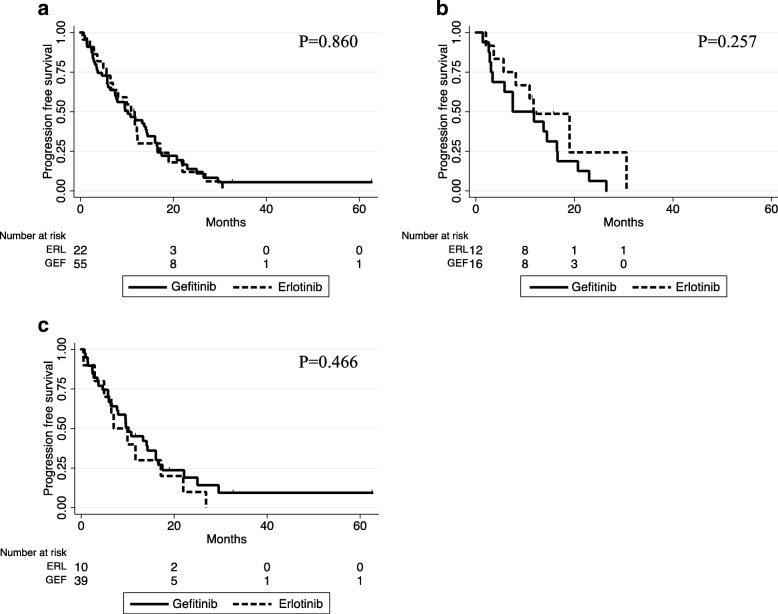
Kaplan-Meier analysis for PFS in patients treated with gefitinib or erlotinib. **a** All patients in this study. **b** Patients who had brain metastasis before EGFR-TKI administration. **c** Patients who had no brain metastasis before EGFR-TKI administration

Kaplan-Meier plots for PFS are shown in Fig. 1. The median PFS of patients in the erlotinib and gefitinib groups were 11.1 and 9.6 months, respectively (*p* = 0.860, Fig. 1a). Among patients with brain metastasis before EGFR-TKI administration, the median PFS of patients in the erlotinib and gefitinib groups were 11.5 and 9.7 months, respectively (*p* = 0.257, Fig. 1b). Among the patients without brain metastasis, the median PFS of patients in the erlotinib and gefitinib groups were 8.5 and 9.6 months, respectively (*P* = 0.466, Fig. 1c). While there was no significant difference in PFS between groups in either subset analysis, there was a tendency for a longer PFS in the erlotinib group than in the gefitinib group among the patients with brain metastasis.

### Cumulative incidence of CNS progression

**Fig. 2:**
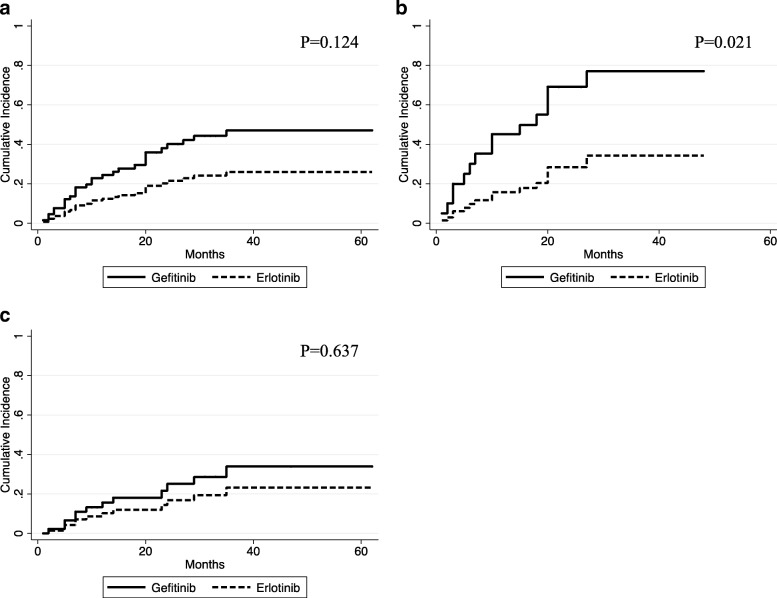
Cumulative incidence of brain metastasis progression using competing risks regression analysis in patients treated with gefitinib or erlotinib. **a** All patients in this study. **b** Patients who had brain metastasis before EGFR-TKI administration. **c** Patients who had no brain metastasis before EGFR-TKI administration

The cumulative incidence curves are shown in Fig. 2. The cumulative risks of CNS progression at 20 and 40 months were 18% and 34%, respectively, in the gefitinib group and 12% and 23%, respectively, in the erlotinib group. The hazard ratio (HR) for the erlotinib group was 0.47 (95% confidence interval [CI], 0.18–1.23; *p* = 0.124). The subgroup analysis showed a significant difference between the erlotinib and gefitinib group among the patients with brain metastasis before EGFR-TKI administration (HR 0.25; 95% CI, 0.08–0.81; *p* = 0.021), while there was no significant difference (HR 0.57; 95% CI, 0.13–3.01; *p* = 0.637) among the patients without brain metastasis.

### Favorable prognostic factors of CNS progression

In the multivariate analysis, the absence of brain metastasis before EGFR-TKI therapy and receiving erlotinib (vs gefitinib) had a significantly favorable effect on CNS progression, while sex, age and ECOG PS had no significant influence. More details are presented in Table 2.

**Table 2:** Multivariate analysis of the clinical characteristics prognostic of central nervous system progression

	HR	95% CI	*P* value
Sex: male vs. female	0.769	0.342–1.729	0.526
Age: ≧70 vs. < 70 years	0.521	0.236–1.150	0.107
ECOG PS: ≧2 vs. < 2	1.013	0.284–3.618	0.984
Brain metastasis: yes vs. no	2.540	1.131–5.702	0.024
EGFR-TKI: erlotinib vs. gefitinib	0.321	0.114–0.903	0.031

In the Cox proportional hazard regression model, the variables adjusted for included sex, age, ECOG PS, presence of brain metastasis at the start of EGFR-TKI treatment, and the EGFR-TKI agent used

*ECOG PS* Eastern Cooperative Oncology Group performance status, *EGFR-TKI* epidermal growth factor receptor tyrosine kinase inhibitor

## Discussion

Several retrospective subset studies indicated that gefitinib was more likely to progress brain metastases in EGFR−mutant advanced NSCLC patients than erlotinib. Omuro et al. reported that 33% of patients treated with gefitinib showed CNS progression as the initial site of progression [11], and Yamamoto et al. reported 3.9% of patients treated with erlotinib showed CNS progression [12]. However, no prospective studies comparing gefitinib with erlotinib has been reported with regard to CNS progression.

In the PFS analysis of our study for patients with brain metastasis, there was a tendency toward a longer PFS in the erlotinib than in the gefitinib group (Fig. 1b). In the cumulative incidence analysis, the probability of CNS progression was lower in the erlotinib group than in the gefitinib group. Particularly, among the patients who had brain metastasis before EGFR-TKI administration, there was a significant difference between the erlotinib and gefitinib groups (Fig. 2b). In the multivariate analysis, we found that receiving erlotinib (vs gefitinib) and absence of CNS metastasis before EGFR-TKI administration are favorable prognostic factor for CNS progression, while sex, age, and ECOG PS had no significant influence on CNS prognosis.

In a randomized phase 3 trial comparing gefitinib and erlotinib efficacy in lung adenocarcinoma patients pretreated with chemotherapy, Urata et al. reported equivalent PFS, overall survival (OS), response rate (RR), and disease control rate (DCR) between gefitinib and erlotinib treatments (8.3 and 10.0 months [HR, 1.093; 95%CI, 0.879 to 1.358; *p* = 0.424], 26.5 and 31.4 months [HR, 1.189; 95%CI, 0.900 to 1.570; *p* = 0.221], 58.9% and 55.0% [*p* = 0.476], and 81.7% and 84.4% [*p* = 0.517], respectively) [13]. The results of our study suggested that erlotinib has better efficacy to control CNS metastasis, and contributes to longer PFS among patients with brain metastasis than gefitinib. The maximum blood concentration and area under the curve were 2120 ng/ml and 38,420 ng/h/ml for an erlotinib dose of 150 mg daily (approved dose in Japan) [14] and 307 ng/ml and 5041 ng/h/ml for a gefitinib dose of 225 mg daily (the approved dose in Japan is 250 mg daily) [15], respectively. Togashi et al. reported that the cerebrospinal fluid concentration and penetration rate of erlotinib (150 mg daily) were significantly higher than those of gefitinib (250 mg daily) [16]. Because of these factors, erlotinib may be superior to gefitinib for controlling CNS metastasis.

Our study has some limitations. Baseline characteristics varied among the study subjects. This difference may have introduced potential bias, which in turn may have affected the study outcomes. First, more patients had brain metastasis in erlotinib group compared with gefitinib group. In the past report, disruption of the blood-brain barrier (BBB) in the presence of CNS metastasis is likely to lead to locally increased drug concentration [17]. Second, more patients had history of radiotherapy for brain metastasis in erlotinib group than gefitinib group. Zeng et al. reported that whole brain radiotherapy (WBRT) combined with an EGFR-TKI increase the BBB permeability of the EGFR-TKI [18]. Magnuson et al. demonstrated a tendency for upfront stereotactic radiosurgery (SRS) or WBRT followed by an EGFR-TKI to decrease intracranial disease progression better than an upfront EGFR-TKI followed by SRS or WBRT [19]. Third, Exon 19 deletion was detected more frequently in erlotinib group than gefitinib group in our study. Lee CK et al. reported that exon 19 deletions were associated with longer PFS than exon 21 L858R substitution in their meta-analysis [20]. Forth, more of the patients who received gefitinib, compared with the erlotinib, had a poor ECOG PS in this study. While few studies have compared PFS and OS after EGFR-TKI treatment between patients with a good PS and those with a poor PS, Kudoh et al. reported that elderly patients with a poor PS are more likely to develop interstitial lung disease than younger patients with a good PS [21]. These differences of baseline might have had a favorable influence on the patients in the erlotinib group of our study.

On the other hand, more patients had history of chemotherapy prior to EGFR-TKI therapy in erlotinib group than gefitinib group. Xu J et al. reported that first-line therapy with EGFR-TKI therapy achieved longer PFS and higher objective response rate (ORR) compared with second line therapy [22]. This factor could have had adverse influence on erlotinib group.

In addition, due to the retrospective nature of the study brain MRI or CT was not performed routinely but only when clinically indicated, which may have affected the evaluation of the time to CNS progression.

## Conclusion

This retrospective study suggested the value of erlotinib as a more promising treatment for patients with EGFR mutant NSCLC with brain metastasis compared with gefitinib. Further pre-planned and large-scale studies are warranted to confirm these results.

## Abbreviations

BBB: Blood-brain barrier
CNS: Central nervous system
CT: Computed tomography
DCR: Disease control rate
ECOG PS: Eastern Cooperative Oncology Group performance status
EGFR-TKI: Epidermal growth factor receptor tyrosine kinase inhibitor
MRI: Magnetic resonance imaging
NSCLC: Non-small-cell lung cancer
ORR: Objective response rate
OS: Overall survival
PFS: Progression free survival
RR: Response rate
SRS: Stereotactic radiosurgery
WBRT: Whole brain radiotherapy
